# One-Leg Standing Test with Eyes Open as a Screening Tool for Prefrailty in Community-Dwelling Older Japanese Women

**DOI:** 10.3390/healthcare12232378

**Published:** 2024-11-26

**Authors:** Zhenyue Liu, Shuji Sawada, Hisashi Naito, Shuichi Machida

**Affiliations:** 1 Graduate School of Health and Sports Science, Juntendo University, Chiba 270-1695, Japan; sh4222011@juntendo.ac.jp (Z.L.); sh-sawada@juntendo.ac.jp (S.S.); hnaitou@juntendo.ac.jp (H.N.); 2 Institute of Health and Sports Science & Medicine, Juntendo University, Chiba 270-1695, Japan; 3 Faculty of Health and Sports Science, Juntendo University, Chiba 270-1695, Japan

**Keywords:** prefrailty, early detection, static balance, community-dwelling older adults

## Abstract

**Background/Objectives**: One-leg standing test with eyes open (OLST), a well-known balance assessment, is simple to implement and requires no special measuring equipment or space. Prefrailty has greater reversibility than frailty, and early detection of prefrailty is essential for frailty prevention in older adults, especially women. However, the association between the OLST and prefrailty remains unclear. Therefore, this study aimed to verify the relationship between the OLST and prefrailty and to validate the effectiveness of the OLST as a screening tool for prefrailty in older Japanese women. **Methods**: This study included 208 community-dwelling older women (mean age: 74.4 ± 5.1 years; range: 65–89) who underwent frailty assessments and OLST. Prefrailty was assessed using the Japanese version of the Cardiovascular Health Study (J-CHS) criteria. The association between prefrailty and OLST was assessed by binary logistic regression analysis, and receiver operating characteristic analyses were performed to examine the effectiveness of OLST as a screening tool for prefrailty. **Results**: OLST time was significantly associated with prefrailty, and those with higher OLST times were less likely to have prefrailty (ORs: 0.98; 95% CI: 0.97–0.99; *p* < 0.001). The area under the curve of the OLST for prefrailty was 0.713 (*p* < 0.001), and the optimal OLST cut-off time for discriminating prefrailty was 24 s (sensitivity: 0.56, specificity: 0.77). **Conclusions**: OLST could be used as a screening tool for prefrailty in older Japanese women. These findings may contribute to the early detection and prevention of frailty.

## 1. Introduction

Frailty is an age-related condition characterized by a decline in physiological function and reserve capability and increased susceptibility to stressors [1,2]. Frailty is associated with adverse outcomes such as falling, poor quality of life, disability, and mortality [1,3] and has become a significant public health issue affecting healthy longevity [4]. The estimated total prevalence of frailty and prefrailty is approximately 50% in the older Japanese population aged ≥65 years, with a greater prevalence in women [5]. Prefrailty, the early stage of frailty, is common in older adults [6]. A recent review and meta-analysis reported that a direct transition from robust to frail or from frail to robust is rare; prefrailty is common between robust and frail, with greater reversibility than frailty [7]. Therefore, early detection of prefrailty and the development of appropriate interventions are essential for frailty prevention and healthy longevity in older adults, especially women.

Of the many frailty assessment methods [8], the Cardiovascular Health Study (CHS) criteria proposed by Fried [9] is the most widely used [10]. Recently, the Japanese version of the CHS (J-CHS) criteria was developed and revised based on the CHS, which has five components, including shrinking, weakness (grip strength), exhaustion, slowness (gait speed), and low activity, which are evaluated through physical fitness tests and questionnaires. The J-CHS criteria is widely used among Japanese older adults as a standard tool for assessing prefrailty and frailty [11,12].

Balance is an important component of physical fitness in older adults [13]. A recent study showed that the presence of postural instability, as assessed by the Berg Balance Scale, determines a greater risk of prefrailty or frailty [14]. Moreover, a greater center of pressure excursion and instability, as assessed by a force platform, was associated with frailty in older adults [15]. In addition, the Timed Up-and-Go test, which assesses dynamic balance and functional mobility, has also been shown to be associated with frailty and has the potential to be used as a frailty identification tool [16,17]. Although previous studies have examined the relationship between frailty status and balance, most of these balance assessments require specialized equipment or space, which may limit their applicability for large-scale use. For early detection and prevention of frailty, it is important to develop a balance measurement tool that is more convenient and accessible for older adults and to examine its relationship with prefrailty.

The one-leg standing test with eyes open (OLST), a well-known balance assessment tool [18], measures the time that an individual can stand on a single lower limb without support to assess postural stability by quantitative measurement [19]. The OLST time begins to decrease sharply in the sixth decade of life [20], and recent studies have reported that an OLST time of <10 s predicted fracture risk in older women [21] and future survival in middle-aged and older adults [22]. The OLST can also be used as a screening tool for low muscle mass [23] and locomotive syndrome [24,25]. Therefore, the OLST time is considered an essential indicator of physical fitness in older adults and requires sufficient attention. However, the association between the OLST and prefrailty, as measured using the J-CHS criteria, remains unclear. Although the J-CHS standard has five components, including grip strength and walking speed, as measures of physical function, it does not include a component of balance. Thus, the relationship between prefrailty and OLST as an assessment of balance requires investigation. Moreover, compared to other assessment tools, the OLST is simple to implement and requires no special measuring equipment or space, which may be more convenient for early screening or self-assessment of prefrailty in large-scale community settings.

Therefore, in the current study, we aimed to verify the relationship between the OLST and prefrailty as evaluated using the J-CHS criteria and to validate the effectiveness of the OLST as a screening tool for prefrailty in older Japanese women. Unlike more comprehensive assessments, OLST offers the advantage of simplicity, which may enhance its applicability in primary care and public health contexts.

## 2. Materials and Methods

### 2.1. Participants

**Figure 1:**
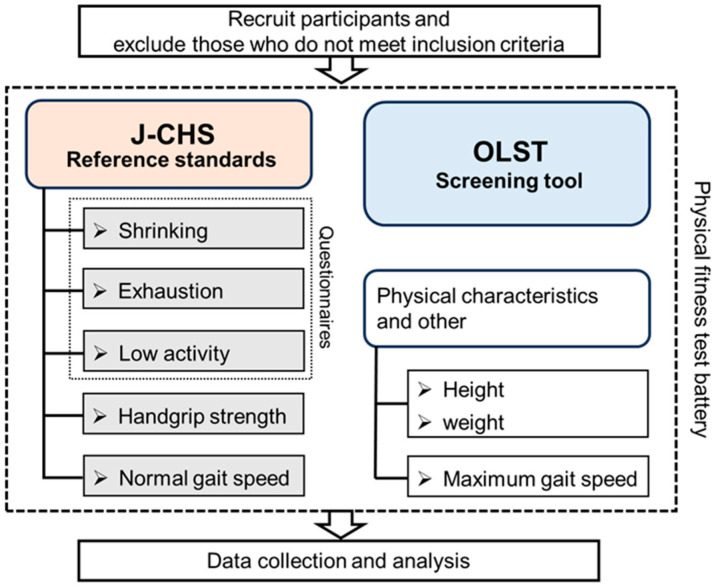
Flowchart of the study protocol. J-CHS, Japanese version of the Cardiovascular Health Study; OLST, one-leg standing test with eyes open.

This cross-sectional study included 208 participants (age: 74.4 ± 5.1 years; range: 65–89). The participants were community-dwelling Japanese women aged ≥65 years living independently. All participants were recruited through printed media in the Chiba Prefecture, Japan. The participants were informed of the study’s methods, procedures, and risks and provided written informed consent before participating. We excluded individuals who did not follow our instructions, those with medical conditions (severe orthopedic and cardiovascular diseases) that the physician-in-charge considered might limit their ability to participate in the test, and those who were unable to complete the measurements and questionnaires or to live independently. Because we aimed to explore prefrailty, individuals with frailty were not included in the analyses. The participant’s physical characteristics, components of the revised J-CHS criteria, and the OLST were evaluated; the study protocol is shown in Figure 1. A priori power analysis was conducted to determine the required sample size, based on an expected effect size of 0.5 (moderate), with 80% power and a significance level of 0.05. This analysis indicated that a minimum of 64 participants in each group would be needed for meaningful statistical comparisons. This study was conducted in accordance with the Declaration of Helsinki and was approved by the Ethics Committee for Human Experiments of Juntendo University (Approval Number: 2022-42; 2022-73).

### 2.2. Reference Standards for Frailty (Revised J-CHS Criteria)

The prefrailty state was assessed according to the revised J-CHS criteria [12]. The J-CHS is based on the Fried phenotype model [9] and includes five components: shrinking, weakness, exhaustion, slowness, and low activity. Weakness and slowness were evaluated by measuring handgrip strength and normal gait speed, respectively, while the other three components were assessed using questionnaires. The details of this process are described below.

Measurements: Handgrip strength was measured using a Smedley-type hand dynamometer (T.K.K.5401; Takei Scientific Instruments Co., Ltd., Niigata, Japan). The participants were instructed to perform the test while standing with their elbows extended, interacting with their right and left hands, and testing each hand twice in four trials. The best of the four tests was used in the analysis, and the cut-off criterion for weakness was defined as <18 kg in women [12]. Normal gait speed was calculated by measuring the time the participants took to walk a 5- or 10-m path on a hard surface at a normal walking speed in daily life. The participants were given acceleration and deceleration sections of 1 or 2 m at the beginning and end of the 5- or 10-m path, respectively. Timing began when the participant’s first foot crossed the starting line and ended when the second foot crossed the finish line. Walking time was measured with a digital stopwatch, and the cut-off criterion for slowness was defined as <1.0 m/s [12].

Questionnaires: Shrinking was assessed using the question “Have you unintentionally lost ≥2 kg in the past 6 months?”, with an answer of “Yes” taken to indicate shrinking. Exhaustion was assessed using the question “In the past 2 weeks, have you felt tired without reason?”, with an answer of “Yes” taken to indicate exhaustion. Low activity was assessed by the questions “Do you engage in moderate levels of physical exercise or sports aimed at health?” or “Do you engage in low levels of physical exercise aimed at health?”, with an answer of “No” to both questions taken to indicate low activity.

If none of the components corresponded, the patient was certified as robust (J-CHS score = 0). If one or two components corresponded, the patient was certified as having prefrailty (J-CHS score = 1 or 2).

### 2.3. One-Leg Standing Test with Eyes Open (OLST)

The participants performed the OLST barefoot with their eyes open on a flat, hard floor and were instructed to decide which leg they would like to stand on based on adequate practice before the test. Two trained examiners stood close to the participants throughout the tests; one acted as a timekeeper using a digital stopwatch, and the other prevented falls or injuries caused by loss of balance. The participants were instructed to stand facing the wall at a distance of >1 m and then stand on the leg they had selected without assistance while keeping their hands on their hips. The test was ended under the following occurs: the raised leg touched the supporting leg or floor, the supporting leg was out of position, and both or one hand was separated from the hip [19]. We set the maximum measurement time to 120 s for each test, and the participants were given two trials unless they could complete 120 s on the first trial. The best of the two trial times was used for the analysis. All tests were conducted under standardized conditions, with verbal instructions provided by trained assessors to ensure consistency across trials. The OLST measurement method in this study was based on that described in the Japan Sports Agency’s “New Physical Fitness Test” implementation guidelines [26].

### 2.4. Other Measurements

The maximum gait speed was measured along with the normal gait speed. The time taken for the participant to walk a 5- or 10-m path at maximum walking movement on hard surfaces was measured using a digital stopwatch. Maximum gait speed was calculated by dividing the walking distance (m) by the walking time (s) at maximum walking movement. Body mass index (BMI) was calculated by dividing body weight (kg) by the square of height (m^2^).

### 2.5. Statistical Analysis

Data are presented as the means and standard deviations (continuous variables) or numbers and percentages (categorical variables). The Shapiro–Wilk test [27] was used to assess the normality of the data from the prefrailty and robust groups. Between-group comparisons were made using the independent-samples *t*-test [28] for continuous variables with a normal distribution (height, weight, and maximum gait speed) or the Mann–Whitney *U* test [29] for continuous variables with a non-normal distribution (age, BMI, handgrip strength, normal gait speed, and OLST). The difference in OLST time distribution between the prefrailty and robust groups was compared using the chi-square test. The association between prefrailty and the OLST was assessed using binary logistic regression analysis. The OLST time, age, BMI, handgrip strength, normal gait speed, and maximum gait speed were included as variables in the regression model, and the unadjusted and adjusted odds ratios (ORs) with their respective 95% confidence intervals (95% CIs) were calculated and used to confirm the significance of the results. To examine the parameters as screening tools for prefrailty, receiver operating characteristic (ROC) analyses were conducted, and the area under the curve (AUC), sensitivity, and specificity were calculated. The optimal cut-off value was derived from the maximum value of the Youden index, which was calculated as Sensitivity + Specificity − 1 [30]. The proportions of J-CHS scores between the two groups, which were divided according to the optimal OLST cut-off time, were compared using the chi-square test. Statistical significance was set at *p* < 0.05, and statistical analyses were conducted using SPSS software (version 29.0.0.0; IBM Japan, Tokyo, Japan).

## 3. Results

The physical characteristics of the participants and the comparison between the prefrailty and robust groups are shown in Table 1. Of the 208 women included in this study, 81 (38.9%) and 127 (61.1%) were in the prefrailty and robust groups, respectively. The component of prefrailty (according to the revised J-CHS criteria) that affected the largest proportion of all participants was exhaustion (n = 44; 21.1%), followed by low activity (n = 20; 9.6%), weakness (n = 19; 9.1%), shrinking (n = 12; 5.8%), and slowness (n = 2; 0.9%).

**Figure 2:**
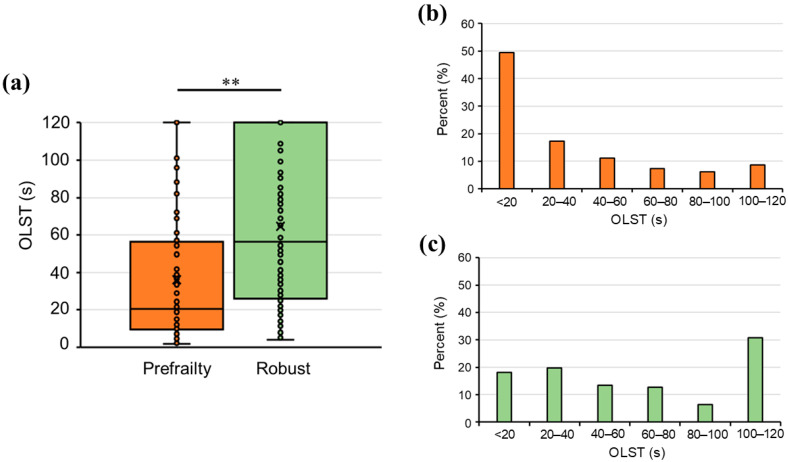
(**a**) Comparison of the OLST between the prefrailty and robust groups. The median and interquartile range of OLST times in the two groups are shown. (**b**) Distribution of the participants in the prefrailty group (n = 81) by OLST times. (**c**) Distribution of the participants in the robust group (n = 127) by OLST times. ** *p* < 0.001; OLST, one-leg standing test with eyes open.

Compared to the robust group, the prefrailty group was significantly older (*p* = 0.028; Table 1) and had lower handgrip strength (*p* < 0.001; Table 1), normal gait speed (*p* < 0.001; Table 1), maximum gait speed (t = 3.556, *p* < 0.001, 95% CI: −0.064–0.223; Table 1), and OLST (*p* < 0.001; Table 1, Figure 2a). No significant differences in height (t = 1.232, *p* = 0.219, 95% CI: −0.522–2.260; Table 1), weight (t = 0.003, *p* = 0.998, 95% CI: −2.042–2.048; Table 1), and BMI (*p* = 0.477; Table 1) were observed between the two groups. Figure 2 shows the distribution of participants across OLST time categories in the prefrailty (Figure 2b) and robust (Figure 2c) groups. There were significant differences in the distribution of individuals across time categories between the two groups, especially in the < 20 s (49.4% vs. 18.1%; Figure 2b,c) and 100–120 s (8.6% vs. 30.7%; Figure 2b,c) categories (χ^2^ = 28.51; df = 5; *p* < 0.001; Figure 2b,c).

The univariate binary logistic regression model (unadjusted model 1; Table 2) showed that the OLST is associated with prefrailty and that a longer OLST time significantly decreased the odds of having prefrailty (ORs: 0.98; 95% CI: 0.97–0.99; *p* < 0.001; unadjusted model 1; Table 2). The effect of OLST after adjusting for age and BMI was unchanged (ORs: 0.98; 95% CI: 0.97–0.99; *p* < 0.001; model 2; Table 2). After adding handgrip strength, normal gait speed, and maximum gait speed to model 2, the OLST (ORs: 0.98; 95% CI: 0.97–0.99; *p* < 0.001; model 3; Table 2), handgrip strength (ORs: 0.83; 95% CI: 0.75–0.92; *p* < 0.001; model 3; Table 2), and normal gait speed (ORs: 0.07; 95% CI: 0.01–0.70; *p* < 0.05; model 3; Table 2) were significantly associated with prefrailty, suggesting that these components were independent predictors of having prefrailty after adjusting for age and BMI.

**Figure 3:**
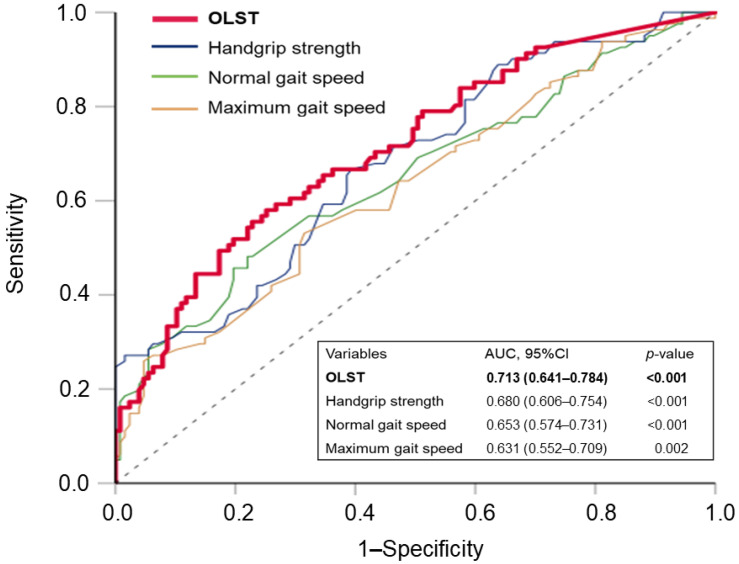
Receiver operating characteristic (ROC) analyses of variables for discriminating prefrailty in participants. AUC, 95% CI, and *p*-values are given. AUC, area under the curve; CI, confidence interval; OLST, one-leg standing test with eyes open.

The ROC analyses are shown in Figure 3, where the AUC of the OLST (0.713; *p* < 0.001), handgrip strength (0.680; *p* < 0.001), normal gait speed (0.653; *p* < 0.001), and maximum gait speed (0.631; *p* = 0.002) for the assessment of prefrailty are indicated. The optimal OLST cut-off time for discriminating prefrailty assessment in older women was 24 s (sensitivity: 0.56; specificity: 0.77), derived from the maximum value of the Youden index.

**Figure 4:**
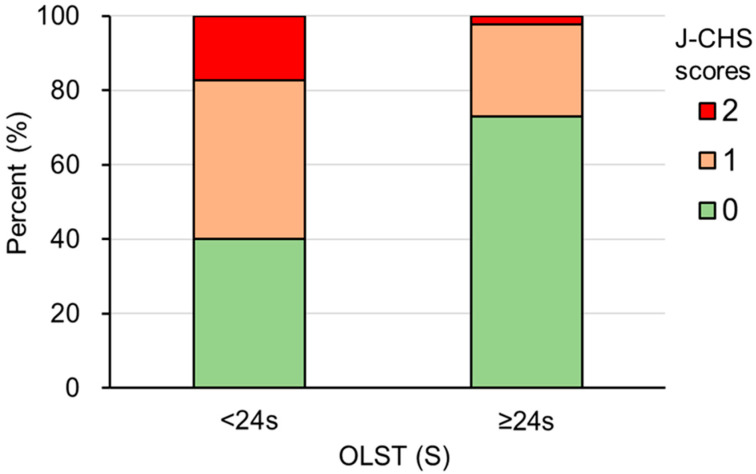
Comparison of the proportion of J-CHS score (0, 1, and 2) in the two groups divided according to the optimal OLST cut-off time (24 s). J-CHS score of 0 is “robust”, and scores of 1 or 2 are “prefrailty”. Proportions between the two groups were compared using the chi-square test. OLST, one-leg standing test with eyes open; J-CHS, Japanese version of the Cardiovascular Health Study.

The proportions of J-CHS scores in the two groups divided according to the optimal OLST cut-off time of 24 s are shown in Figure 4. The results of the chi-square test showed that the proportions of J-CHS scores of 1 and 2 in the <24-s group were 42.7% and 17.3%, respectively, which were higher than those of J-CHS scores of 1 and 2 in the ≥24-s group, which were 24.8% and 2.3%, respectively (χ^2^ = 27.58; df = 2; *p* < 0.001; Figure 4).

## 4. Discussion

In this study, we examined the association between OLST and prefrailty, validated the effectiveness of the OLST as a screening tool for prefrailty as assessed by the J-CHS criteria among healthy older Japanese women, and calculated the cut-off time of OLST at 24 s for prefrailty. To the best of our knowledge, this is the first study to investigate the effectiveness of the OLST as a screening tool for prefrailty. The OLST may be an effective method for screening for prefrailty in community-dwelling, healthy older Japanese women, which may contribute to early screening and intervention for frailty prevention.

In the present study, the prevalence of prefrailty was 38.9%. Regarding the prevalence of the components included in the J-CHS criteria, exhaustion (21.2%), low activity (9.6%), and weakness (9.1%) were relatively common, whereas slowness (0.9%) was nearly absent. In a previous study that evaluated prefrailty using the J-CHS criteria among 352 healthy older Japanese women, the prevalence of prefrailty was 46.6%, and the components of exhaustion (19.6%), low physical activity (21.9%), and weakness (10.5%) were higher, but the component of slowness was lower (0.9%) [31]. These results are similar to those of the current study. Although research on the prevalence of prefrailty and its components is lacking, the results of existing studies suggest that prefrailty in community-dwelling healthy older Japanese women may be characterized by the high probability of subjective fatigue, low physical activity, or low muscle strength but with the extent of mobility maintained.

The participants in the prefrailty group were older and had lower grip strength and gait speed than those in the robust group, which is consistent with the results of a previous study [6]. OLST time also differed between the two groups, and the OLST was independently associated with prefrailty, showing that every 1-s decrease in OLST time increased the probability of prefrailty by 2%. The OLST time decreases with age, especially after 60 years, where it declines by approximately one-half every decade [20,23,24], while the prevalence of prefrailty increases with age by about 10% per decade [5]. This might explain our finding that, the shorter the OLST, the higher the risk of prefrailty.

Our findings show that the OLST can effectively screen for prefrailty in older women, with an optimal cut-off time of 24 s. These findings align with previous studies highlighting the role of balance assessments in predicting prefrailty and frailty risk [14,15]. Moreover, previous studies have reported that the OLST time can be used as a screening tool for locomotive syndrome [24,25,31,32] with a cut-off time of 9 to 26 s (averaged over legs of two sides) and low muscle mass [23] with a cut-off time of 55 s (sum of legs of two sides). The OLST measurement methods used in previous studies have varied, with most studies adopting a measurement of the OLST in both legs for a maximum of 60 s per test. In the current study, we referred to the test methodology of the “new physical fitness tests” released by the Japan Sports Agency, increased the measurement time to 120 s, and measured the OLST of only one leg (chosen by each participant after practicing). Our method may have a smaller ceiling effect, be easier to implement, and be more suitable for early screening and self-diagnosis in older adults, as they can perform this simple test anytime and anywhere. In addition, as a quantifiable measure of balance using time as the unit of measurement, the OLST could also be used as a test to evaluate the effectiveness of exercise interventions. Therefore, it is possible to consider the use of OLST for primary screening and subsequent intervention in large populations. For example, after primary screening with the OLST, the population could be stratified using a 24-s cut-off, and then, exercise interventions of appropriate intensity could be delivered separately to different populations; after which, the OLST would be used for post-intervention reassessment. This could have important implications for improving and preventing frailty in community-dwelling older adults.

It is also notable that the proportion of participants with a J-CHS score of 2 was much higher in the <24-s group than in the >24-s group (17.3% vs. 2.3%), suggesting that the sensitivity of OLST identification may be higher for higher J-CHS scores. Although a previous study showed an association between OLST <5-s and frailty [33], it did not use the OLST quantified in terms of time; therefore, we expect a higher diagnostic validity of the OLST for frailty, which needs to be explored in future studies.

This study has several limitations that warrant discussion. First, the sample size was small and limited to older women, and the participants maintained their mobility and did not include patients who were hospitalized, institutionalized, or receiving nursing care. Thus, our results may be more applicable to healthy older women living in the community who can live independently. Future research is needed to verify its applicability in different populations, including men and non-Japanese older adults, which would help verify its broader utility. Secondly, we did not examine the test–retest reliability and interrater reliability of the OLST, although these values have been reported to be acceptable in previous studies [19]. As the OLST is one of the “new physical fitness tests” released by the Japan Sports Agency, we referred to the test methodology. Thirdly, as this was a cross-sectional study, a causal relationship between OLST and frailty cannot be established. Future research is needed to investigate the longitudinal usefulness of the OLST in predicting transitions between frailty status. Finally, this study did not include other factors related to balance besides the OLST, such as the parameter related to the center of pressure excursion measured by the force platform, and other potentially relevant variables, such as cognitive function or comorbidities, which may influence OLST performance. We focused more on the OLST as a simple physical function test that does not require any special instruments and could be self-assessed by older adults; thus, the convenience of implementation may be more important when considering applications in large-scale screening assessments.

## 5. Conclusions

Our results indicated a clear association between the OLST and prefrailty as evaluated using the J-CHS criteria. The OLST could be used as a screening tool for prefrailty in healthy older Japanese women, with an optimal cut-off time of 24 s, which may contribute to the early detection and prevention of frailty. Its practicality and cost-effectiveness make it a valuable addition to primary care and public health screening programs. Future research is needed to verify the effectiveness of OLST in different populations and in individuals with varying frailty status and to investigate the causal relationship between OLST and frailty through longitudinal studies.

## Figures and Tables

**Table 1:** Participant characteristics and comparison of the parameters between the prefrailty and robust groups.

	Total (n = 208)	Prefrailty (n = 81, 38.9%)	Robust (n = 127, 61.1%)	*p*-Value
Age (year)	74.4 ± 5.1	75.3 ± 5.1	73.8 ± 5.0	0.028 ^a^
Height (cm)	152.1 ± 5.0	151.6 ± 4.8	152.4 ± 5.1	0.219 ^b^
Weight (kg)	51.5 ± 7.3	51.5 ± 6.5	51.5 ± 7.8	0.998 ^b^
BMI (kg/m^2^)	22.3 ± 2.9	22.4 ± 2.7	22.2 ± 3.1	0.477 ^a^
Handgrip strength (kg)	23.3 ± 3.9	21.7 ± 3.9	24.4 ± 3.5	<0.001 ^a^
Normal gait speed (m/s)	1.48 ± 0.22	1.40 ± 0.22	1.53 ± 0.21	<0.001 ^a^
Maximum gait speed (m/s)	1.96 ± 0.29	1.87 ± 0.32	2.02 ± 0.26	<0.001 ^b^
OLST (s)	53.5 ± 41.9	36.0 ± 35.3	64.7 ± 42.0	<0.001 ^a^

Data are presented as the mean ± standard deviation. BMI, body mass index; OLST, one-leg standing test with eyes open. ^a^ Mann–Whitney *U* test; ^b^ Independent-samples *t*-test.

**Table 2:** Univariate and multivariate binary logistic regression assessing the association between OLST and prefrailty.

	Unadjusted Model 1	Model 2 ^a^	Model 3 ^b^
OLST	0.98 ** [0.97, 0.99]	0.98 ** [0.97, 0.99]	0.98 ** [0.97, 0.99]
Age		1.01 [0.95, 1.08]	0.95 [0.89, 1.03]
BMI		0.99 [0.89, 1.10]	1.00 [0.89, 1.12]
Handgrip strength			0.83 ** [0.75, 0.92]
Normal gait speed			0.07 * [0.01, 0.70]
Maximum gait speed			2.35 [0.44, 12.49]

Data are presented as ORs and 95% CI. ORs, odds ratios; CI, confidence interval; OLST, one-leg standing test with eyes open; BMI, body mass index; * *p* < 0.05, ** *p* < 0.001. ^a^ The OLST time, age, and BMI are included as variables in regression model 2. ^b^ The OLST time, age, BMI, handgrip strength, normal gait speed, and maximum gait speed are included as variables in regression model 3.

## Data Availability

The data that support the findings of this study are available from the corresponding author upon reasonable request.
